# Iodine-Induced Retinopathy: A Case Report

**DOI:** 10.4274/tjo.galenos.2020.02686

**Published:** 2020-08-26

**Authors:** Sabiha Güngör Kobat, Fatih Cem Gül, Burak Turgut

**Affiliations:** 1 University of Health Sciences Turkey Faculty of Medicine, Department of Ophthalmology, Elazığ, Turkey; 2 Onsekiz Mart University Faculty of Medicine, Department of Ophthalmology, Çanakkale, Turkey

**Keywords:** Iodine, toxic, retinopathy

## Abstract

Potassium iodide is used as an iodine supplement in salt as part of a national program in Turkey. An overdose of iodine has a toxic effect on the retinal pigment epithelium and photoreceptors. The case presented here is a patient who developed retinopathy following consumption of an excessive dose of iodine.

## Introduction

Potassium iodide is used as an iodine supplement in salt as part of a national program in Turkey. Iodine deficiency is a serious and preventable global healthcare problem that causes several diseases and conditions such as mental retardation, developmental growth retardation, miscarriage, deafness, and goiter. A normal person requires 100-150 µg/day of iodine for developmental growth.^1^ The United Nations Children’s Fund, International Council for Control of Iodine Deficiency Disorders, and World Health Organization recommend national programs for the use of iodized table salt to eliminate iodine deficiency.^2^ The production of iodized table salt became mandatory in Turkey in 1998. However, iodine overdose has a toxic effect on the retinal pigment epithelium (RPE) and photoreceptors. In this report, we present a patient who developed retinopathy following consumption of an excessive dose of iodine.

## Case Report

**Figure 1:**
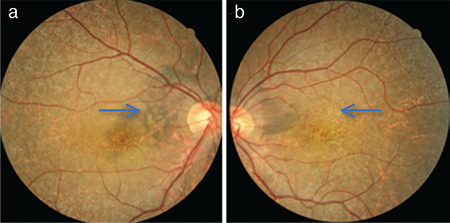
Fundus photograph at initial presentation (20 days after iodine intake) shows hyperpigmentation (arrow) in the right eye (a) and hypopigmentation (arrow) in the left eye (b)

**Figure 2:**
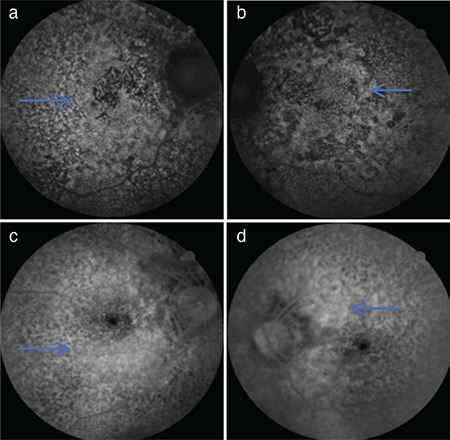
Fundus autofluorescence images shows hyperautofluorescence (arrows) in the right eye (a) and left eye (b). Fundus fluorescein angiography images show widespread window defect (arrows) in the right eye (c) and left eye (d)

**Figure 3:**
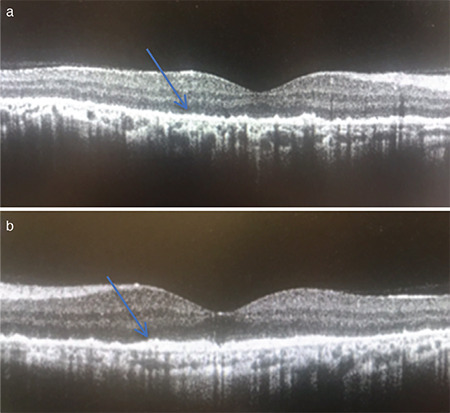
OCT images show hyperreflective points (arrows) on the RPE in the right eye (a) and left eye (b) OCT: Optical coherence tomography

A 39-year-old man presented to the outpatient clinic with sudden vision loss that had started 15 days previously. His visual acuity in both eyes was at the level of hand motion. Pupillary light reflexes and eye movements were normal. Anterior segment biomicroscopy findings and intraocular pressure were normal. On dilated fundus examination, hypopigmentation was present in the retina and macula, as well as points of hyperpigmentation. At initial presentation, the hyperpigmentation was mainly around the disc and in the peripheral retina (Figure 1a, b). Intense points of hyperfluorescence were present in fundus autofluorescence (Figure 2a, b). Widespread window defect was seen on angiography (Figure 2c, d). Hyperreflective points of accumulation were detected over the RPE on optical coherence tomography (Figure 3a, b). Visual-evoked potential testing was requested for the patient and showed prolonged P100 wave latencies in both eyes.

**Figure 4:**
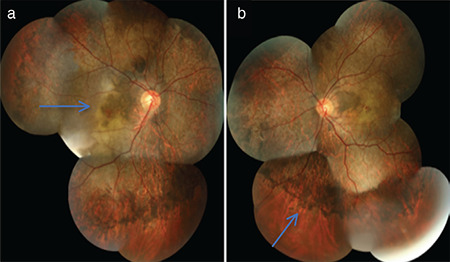
Fundus photographs at 3 months after iodine intake show hyperpigmentation around the disc and hypopigmentation (arrow) in the right eye (a) and hyperpigmentation in the peripheral retina of the left eye (b)

The patient was taking antidepressants for psychiatric reasons and was reluctant to give a detailed history. There was no remarkable family history and when the patient was questioned in detail, it was learned that he worked in a salt factory and had attempted suicide with iodine approximately 20 days earlier because of psychological problems. Although the patient could not remember the exact amount, he estimated that he had consumed 200 mL of mildly diluted iodine. The patient reported color vision disruption starting 2-3 days after this event, followed by vision loss. Thyroid function tests, complete blood count, and biochemical examinations were requested. The T3, T4, and thyroid stimulating hormone levels were normal. Treatment with vitamin B and micronutrient supplements was started and follow-up was scheduled for 1 month later. In the follow-up examination, vision was at the level of counting fingers from approximately 1 meter and color vision was still impaired. On fundus examination, there was an appearance of increased hyperpigmentation, especially around the disc and in the peripheral retina at 2 months after iodine intake. The findings persisted 1 month later. Visual acuity was at the level of 0.1 (-1.75) Snellen lines in the right eye and counting fingers from 1 meter in the left eye. Hyperpigmentation around the disc was determined to have advanced and choroideremia had developed in the right eye, and pigmentation had become evident around the disc and in the peripheral retina in the left eye at 3 months after iodine intake (Figure 4a, b. Visual field examination was performed and there was determined to be absolute scotoma in both eyes.

## Discussion

 Potassium iodide is a water-soluble salt that is rapidly absorbed after consumption. When mixed with blood, iodide is metabolized within 2-3 minutes by glutathione in erythrocytes.^3^ Iodide is received by the thyroid gland and is used in the synthesis of thyroid hormones. Iodine intake was shown in the thyroid gland, kidneys, liver, and eyes. In a previous study, iodine was seen in the vitreous of rabbits within 3-6 hours of Iodine-131 injection. The tissues taking in iodine are the RPE and choroid in particular.^4^ Animal studies have reported that a dose of 20-30 mg/kg is toxic for the retina.^5^ Several studies have used iodate to induce damage to photoreceptor cells and the RPE.^6^ It has been understood that the RPE is affected in particular by iodate toxicity.^7^ In animal models of iodate-induced retinal toxicity, changes similar to those of dry-type age-related macular degeneration have been seen histopathologically.^8^

The first cases of retinal iodate toxicity, reported by Schimmel and Riehm in 1926, were 2 cases of blindness which developed following an injection of Septojod, which contained sodium hypoiodate and sodium hypoiodide as an antibacterial agent.^9^ The most extensive case series was of 5 patients reported by Singalavanija et al.^3^

Previous studies have determined that iodate toxicity varied depending on the amount of iodate intake.^10^ Following iodate intake, the liver, kidneys, gastrointestinal system mucosa, bladder, and retina are affected.^10^ While the damage to other organs is reversible, retinal damage has been determined to be irreversible.^11^

In the current case, vision symptoms started approximately 5 days after the intake of potassium iodide and progressively worsened to a maximum reduction in sight at approximately 20 days. However, as a result of probable photoreceptor regeneration during the follow-up period, visual acuity showed an improvement up to the level of 0.1.

In cases of iodide intoxication, the first treatment is emesis induction and gastric lavage to prevent absorption. However, if iodine has been absorbed, the patient must be closely monitored with observation of fluid and electrolyte imbalances, and treatment must be planned according to the organs affected.^12^ In the case series of 5 patients by Singalavanija et al.^3^, the patients were administered prednisolone and vitamins B1, B6, and B12. It was concluded that the visual recovery obtained in treatment was dependent on the amount of iodate consumed. In the current patient, no systemic pathology was determined and the retinal toxicity was followed up with the administration of vitamin supplementation.

In conclusion, iodide is a compound which has a dose-dependent toxic effect on the retina, and although there have been a few case series of retinal toxicity, no effective treatment has been determined. Care must be taken with individuals with oral intake >187 mg/kg, as the possibility of retinal toxicity is high. There is a need for further more extensive series and animal studies to understand the mechanism of iodine toxicity and to develop treatments.
